# Integral T-Shaped Phantom-Dosimeter System to Measure Transverse and Longitudinal Dose Distributions Simultaneously for Stereotactic Radiosurgery Dosimetry

**DOI:** 10.3390/s120506404

**Published:** 2012-05-14

**Authors:** Wook Jae Yoo, Jinsoo Moon, Kyoung Won Jang, Ki-Tek Han, Sang Hun Shin, Dayeong Jeon, Jang-Yeon Park, Byung Gi Park, Bongsoo Lee

**Affiliations:** 1 School of Biomedical Engineering, College of Biomedical & Health Science, Research Institute of Biomedical Engineering, Konkuk University, 322 Danwol-dong, Chungju-si, Chungcheongbuk-do, 380-701, Korea; E-Mails: wonzip@naver.com (W.J.Y.); mjs092082@nate.com (J.M.); kko988@hotmail.com (K.W.J.); nikev2@nate.com (K.-T.H.); shshin9431@gmail.com (S.H.S.); jdy603@naver.com (D.J.); jyparu@kku.ac.kr (J.-Y.P.); 2 Department of Energy & Environment Engineering, College of Engineering, Soonchunhyang University, 646 Eupnae-ri, Shinchang-myeon, Asan-si, Chungcheongnam-do, 336-745, Korea; E-Mail: byunggi@sch.ac.kr

**Keywords:** fiber-optic dosimeter, phantom, absorbed dose, square scintillating optical fiber, radiosurgery

## Abstract

A T-shaped fiber-optic phantom-dosimeter system was developed using square scintillating optical fibers, a lens system, and a CMOS image camera. Images of scintillating light were used to simultaneously measure the transverse and longitudinal distributions of absorbed dose of a 6 MV photon beam with field sizes of 1 × 1 and 3 × 3 cm^2^. Each optical fiber has a very small sensitive volume and the sensitive material is water equivalent. This allows the measurements of cross-beam profile as well as the percentage depth dose of small field sizes. In the case of transverse dose distribution, the measured beam profiles were gradually become uneven and the beam edge had a gentle slope with increasing depth of the PMMA phantom. In addition, the maximum dose values of longitudinal dose distribution for 6 MV photon beam with field sizes of 1 × 1 and 3 × 3 cm^2^ were found to be at a depth of approximately 15 mm and the percentage depth dose of both field sizes were nearly in agreement at the skin dose level. Based on the results of this study, it is anticipated that an all-in-one phantom-dosimeter can be developed to accurately measure beam profiles and dose distribution in a small irradiation fields prior to carrying out stereotactic radiosurgery.

## 1. Introduction

As a medical procedure that allows treatment of the brain, spinal cord, or certain small tumors, stereotactic radiosurgery is routinely used to treat small brain tumors that are typically 3 cm or less in diameter. Accordingly, stereotactic radiosurgery requires a precisely focused, high-dose radiation beam that is delivered to a small tumor [1,2]. As the radiation source, three types of equipment are generally used to deliver a therapeutic radiation beam: gamma knife, linear accelerator (LINAC)-based therapy, and proton beam therapy; however, a clinical LINAC is usually used to irradiate a high-energy radiation beam.

In order to perform optimum stereotactic radiosurgery, it is necessary to plan personalized treatment for the area to be treated, the dose of radiation to be given, and accurate dose distributions such as skin, transverse, and depth doses [3–5]. Therefore, the real time, high resolution, and multi-dimensional measurements of the absorbed dose distributions are required for determination of the exact beam to be used for radiosurgery in small target area [6]. Nowadays, many kinds of conventional dosimeters have been developed and used for measurement of the absorbed dose in radiosurgery, such as an ionization chamber, a radiochromic dosimetry film, and a thermoluminescent dosimeter (TLD) [7,8]. However, the aforementioned conventional dosimeters have some problems regarding application to stereotactic radiosurgery dosimetry stemming from their sensitive materials not being water equivalent, which is very important to precisely measure the beam conditions and dose distribution without any corrections. Since the sensing materials of the ionization chamber are different from those of the human body or the water phantom, complicated calibration processes are required to convert readouts to absorbed doses [2,9]. In addition, due to loss of lateral electronic equilibrium and volume averaging, the ionization chamber is inappropriate for use as the dosimeter in stereotactic radiosurgery carried out to tumors and other abnormalities of the brain with a small beam field of 1 × 1 ∼ 3 × 3 cm^2^. Meanwhile, radiochromic film and TLD require time-consuming reading processes after irradiation, and the consequent lack of real-time measurement capability inhibits accurate dose measurement [4,10]. Furthermore, existing dosimeters need the use of ancillary water or solid-water stack phantoms for measuring dose distributions [10–12].

To overcome these problems, it is necessary to develop a new type of dosimeter that offers water equivalence, multi-channel and real time monitoring, and a small sensing volume for high-resolution and accurate measurement [13]. In this study, therefore, we developed an all-in-one phantom and dosimeter system using scintillating fiber and plastic optical fiber array. The scintillating fiber array in this novel integral phantom-dosimeter system for measuring a high-dose photon beam can minimize perturbation of photon fluence because the sensitive volume and the gap between the sensing elements are very small and the employed sensitive materials have water equivalence. Generally, it is well known that the water-equivalent fiber-optic dosimeters using a scintillating fiber have many favorable dosimetric characteristics, such as their linear response to dose and dose rate, energy independence, and no corrections for temperature, pressure and humidity [14–17].

## 2. Materials and Experimental Setup

### 2.1. Fiber-Optic Phantom-Dosimeter System

In this study, we constructed a T-shaped fiber-optic phantom-dosimeter system that consists of square scintillating optical fibers, a lens system, and a complementary metal-oxide semiconductor (CMOS) image camera. In developing the fiber-optic phantom, a T-shaped optical fiber array is fabricated using two types of square optical fibers. First, square-type scintillating fiber (Saint-Gobain Ceramic & Plastics, BCF-12) is selected as a sensing element of the phantom-dosimeter. The squre type scintillating fiber has a core/single-clad structure with a 1 × 1 mm^2^ rectangular cross-section and a material density of 1.05. The core of the square-type scintillating fiber was sysnthesized with polystyrene (PS) and fluorescent dopants. The thikness of the polymethyl methacrylate (PMMA)-based cladding is approximately 4% of the fiber size. This organic scintillator yields about 8,000 photons per MeV from an ionizing particle and the decay time is 3.2 ns. The emission color and the peak wavelength of generated scintillating light are blue and 435 nm, respectively. Second, square-type transparent optical fiber (Saint-Gobain Ceramic & Plastics, BCF-98) is used to transmit the scintillating light from a scintillating fiber to a light-measuring device, as a clear waveguide. This square-type plastic fiber has a 1 × 1 mm^2^ rectangular cross-section equal to the square-type scintillating fiber, and the materials of the core and the cladding are PS and PMMA, respectively. The refractive indices of the core and the cladding are 1.60 and 1.49, respectively, and the numerical aperture (NA) is 0.58.

**Figure 1.**
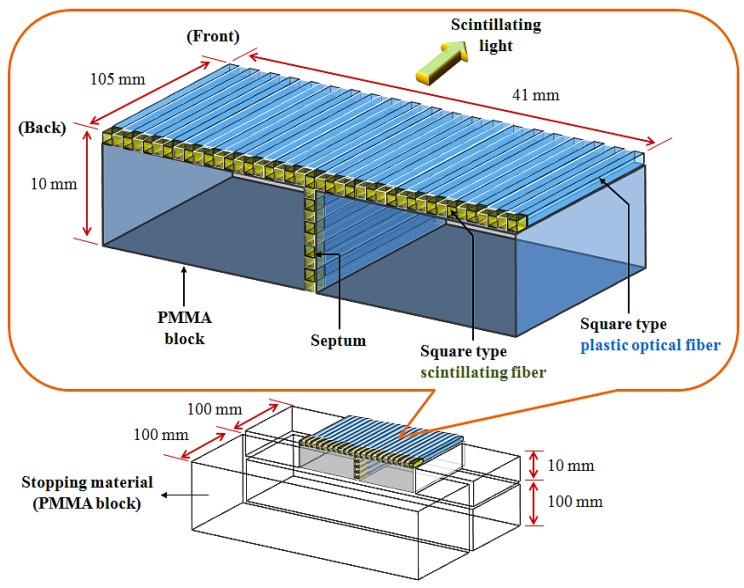
Structure of the fiber-optic phantom.

Figure 1 illustrates the structure of the fiber-optic phantom, which consists of a T-shaped optical fiber array and PMMA blocks. In order to fabricate the T-shaped optical fiber array, a scintillating fiber with a length of 5 mm was polished and connected with optical epoxy (3M, DP-100) to the distal end of a plastic optical fiber whose length is 100 mm. The optical fiber array was then constructed in a T-shape using 50 square fibers that are connected to the scintillating fibers and plastic optical fibers. The gaps between the square fibers were coated by black polyvinyl chloride (PVC) thin films, as septa, to minimize the scintillating light interference among the closely packed square fibers due to the cross-talk effect. Finally, the fiber-optic phantom was assembled with the T-shaped optical fiber array and the PMMA blocks as a frame, and the outer surface of the fiber-optic phantom was covered with a black PVC film to intercept the ambient light noise from the measuring environment. In addition, the extra PMMA blocks were also used as a stopping material and they are placed at the both sides and the bottom of the fiber-optic phantom to provide charge particle equilibrium with respect to the surface of the scintillating fibers, as shown in Figure 1. However, the total volume of the fabricated fiber-optic phantom is small for measuring depth dose with a high-dose photon beam. Furthermore, the fiber-optic phantom consists of a T-shaped optical fiber array and PMMA blocks instead of only an optical fiber array. Therefore, we additionally used a PMMA stack phantom, which was placed on the fiber-optic phantom, for measuring percentage depth dose (PDD) [10].

**Figure 2.**
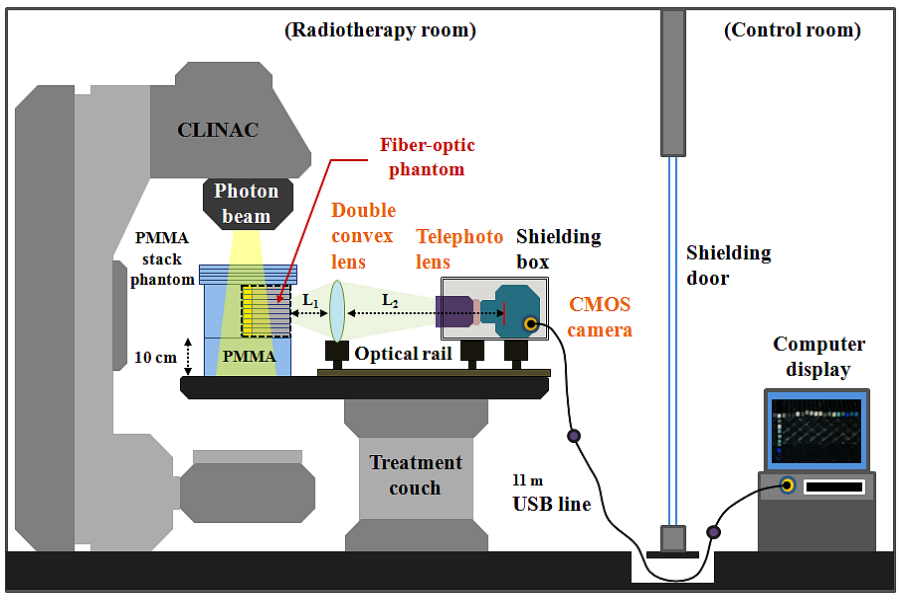
Experimental setup using the proposed T-shaped fiber-optic phantom-dosimeter system.

The experimental setup employed for measuring the scintillating light distribution using the T-shaped fiber-optic phantom-dosimeter system is shown in Figure 2. In order to guide the scintillating light image from a fiber-optic phantom to a complementary metal-oxide semiconductor (CMOS) image camera (Edmund Optics, EO-5012C), a lens system is constructed using a double convex lens (Daeduk Optical Instruments, DCX 0504) and a telephoto lens (Asahi Optics, smc-PENTAX-M 1:2 85 mm). The material of double convex lens is a BK7 grade A optical glass. The diameter and the thickness of a double convex lens are 50 mm and 5.4 mm, respectively, and the focus length of this lens is 250 mm. In the case of an 85 mm telephoto lens, it is mounted with a CMOS camera using a PK-C camera adapter-ring (Shanghai Transvision Photographic Equipment, KIPON K6517) and the aperture of telephoto lens is set to be F 4.0 when taking pictures. As shown in Figure 2, the double convex lens and the CMOS camera having a telephoto lens are equipped at the carriers on the optical rail (Daeduk Optical Instruments, DOI-POR-1000). In this lens system, the distance L_1_ between the back end of fiber-optic phantom and the curved surface of double convex lens is 10.5 cm and the distance L_2_ between the curved surface of double convex lens and the CMOS element of camera is fixed at 90 cm. For generating scintillating light, the center of T-shaped scintillating fiber array in a PMMA stack phantom is accurately located at the isocenter of a clinical LINAC (Varian Medical Systems, CLINAC^®^ 1800) system using a laser alignment system. Therefore, the total distance between the distal end of scintillating fiber and the CMOS element of camera is about 111 cm. In this experiment, a geminated shielding box, which made of aluminum (outside) and lead (inside), is used to protect the CMOS camera from the effects of scattered radiation.

### 2.2. Testing of the System in Cross-Beam Profile and Depth Dose Measurements in Small Fields

In this study, high-resolution and real-time scintillating light images are obtained using the T-shaped fiber-optic phantom-dosimeter system according to the field size and different depths of a PMMA phantom irradiated by a 6 MV photon beam of a CLINAC. The source to surface distance (SSD) is set to 100 cm as the condition for calibrating the CLINAC and the beam field-sizes are set to 1 × 1 and 3 × 3 cm^2^, such as those used in stereotactic radiosurgery. The generated scintillating light due to interactions between a 6 MV photon beam and an organic scintillating fiber array in a fiber-optic phantom that is placed on a treatment couch is guided to the other side of the fiber-optic phantom via a plastic optical fiber array. The scintillating light image is then magnified by a double convex and telephoto lens system and is converted to an electric signal by a CMOS camera. The output image signal generated from the CMOS camera is transmitted by an 11 m universal serial bus (USB) line and connected to a desktop computer in a control room. Finally, the intensities of the scintillating lights transmitted from each optical fiber are analyzed using a MATLAB program (MATLAB^®^, Math Works) for obtaining the dose distributions and beam profile.

**Figure 3.**
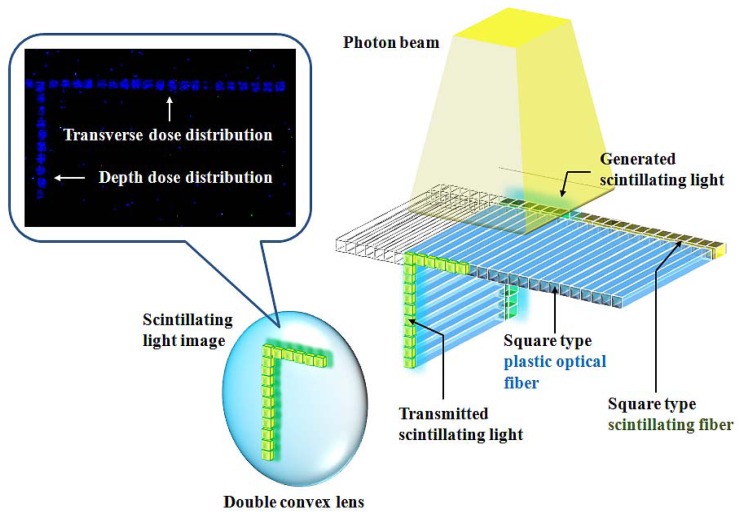
Scintillating light image of the fiber-optic phantom.

Figure 3 shows the acquisition process of a scintillating light image generated from the fiber-optic phantom when the photon beam is irradiated on the center of the T-shaped scintillating fiber array. By a MATLAB program, first, a video file having many frames is read and converted to red-green-blue (RGB) color image files. Second, a RGB scale of the image files is changed to a grey scale. Third, the positions of each scintillating light generated from the scintillating fiber array are selected and the light intensities are calculated. Finally, the light intensity values obtained in many images are calculated the average.

In this experiment, the beam profile was measured on one side from the center of the T array because the cross-beam profile generally has a symmetrical distribution over given beam field by a radially symmetric conical flattening filter in a CLINAC. However, the entire cross-beam profile, which includes the both sides from the center of the fiber-optic phantom, is also measurable by changing the lens array. The images of the scintillating light distribution obtained by the phantom-dosimeter system can be easily and effectively used for calibrating the conditions of the high-dose photon beam in radiosurgery. Furthermore, the transverse dose distributions at specific depths including the skin layer can be obtained using these scintillating light images. In this study, we also obtained PDD according to the depth variation using the average value of the light intensity.

## 3. Results

**Figure 4.**
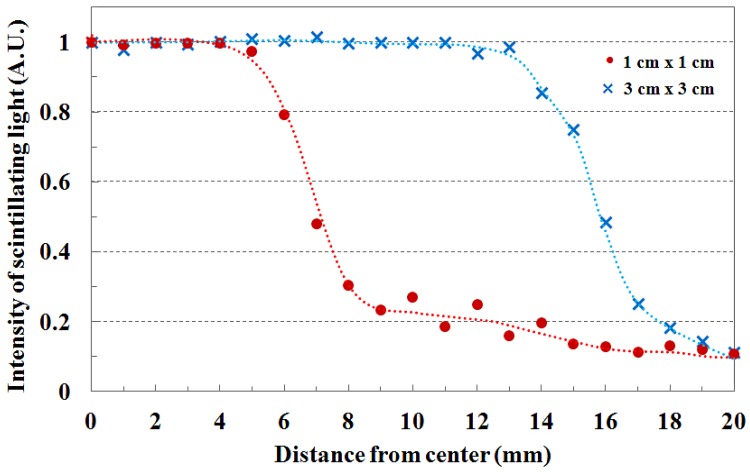
Beam profile and beam edge according to the field size variation at 15 mm depth.

The beam profile and the beam edge according to the field size are shown in Figure 4. When the PMMA stack phantom with 15 mm thickness was placed on the fiber-optic phantom, the scintillating light images of the transverse fiber-optic array about the beam profile were obtained according to two beam field-sizes of 1 × 1 and 3 × 3 cm^2^. Generally, the cross-beam characteristics are affected by the radially symmetric conical flattening filter and the beam transmitted through the flattening filter in the treatment head is flattened by differentially absorbing photons. Therefore, the measured light signals are distributed uniformly over given field-sizes as shown in Figure 4. For accurate cross-beam measurements, the detector size is very important. In the off-axis region, the measured light signal shows a fairly sharp falloff, but it does not show a perfect perpendicular beam edge because it is affected by the penumbra, scattered radiation, and the size of the detector. If the detector size is smaller than the proposed dosimeter, the slope of the measured beam edge is steeper than that of Figure 4 [3,10].

**Figure 5.**
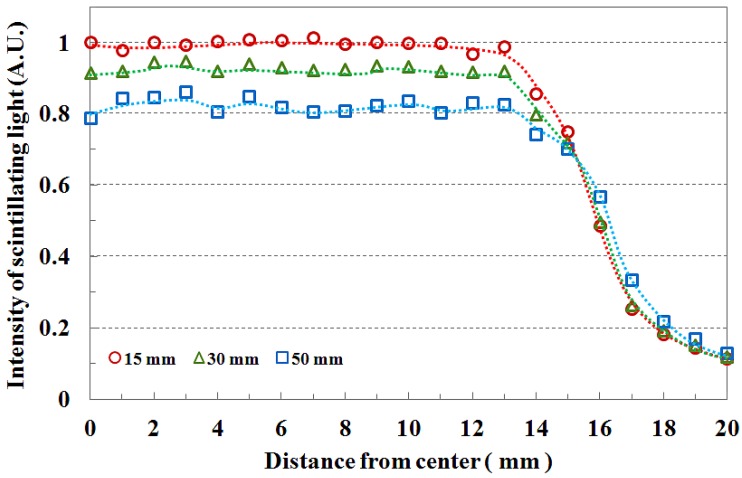
Transverse dose distribution of 3 × 3 cm^2^ field size according to the depth.

Figure 5 shows the field flatness and the transverse dose distribution of 3 × 3 cm^2^ field size according to the depth. The beam field flatness changes with depth. At 15 mm depth, the measured light signals of the transverse fiber-optic array show similar output values. However, the transverse dose distributions gradually become uneven with increasing depth of the PMMA phantom, as shown in Figure 5. This phenomenon is attributed to an increase in scattering of the primary dose ratio in accordance with increasing depth of the human body or phantom and decreasing incident photon energy off-axis. In Figure 5, the measured scintillating light signals about the beam edge also have a gentle slope with increasing depth due to the penumbra region.

**Figure 6.**
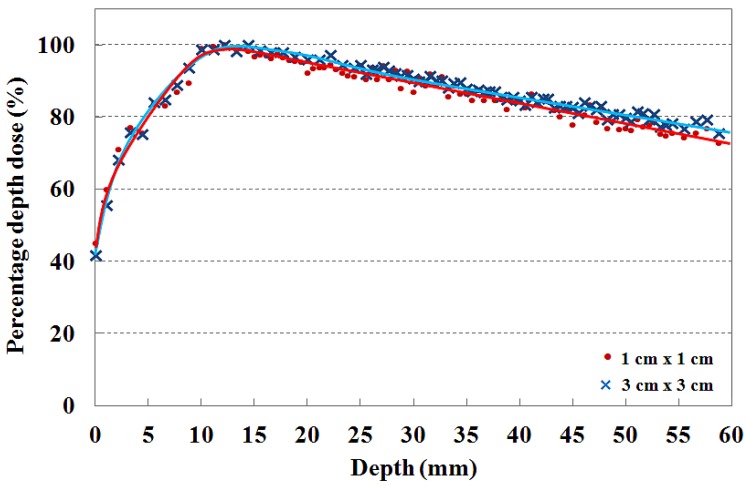
Measurement of percentage depth doses with 1 × 1 and 3 × 3 cm^2^ field sizes.

The PDDs that are measured by using the T-shaped fiber-optic phantom-dosimeter with a 6 MV photon beam are shown in Figure 6. In clinical practice, the central axis dose distribution is characterized by the PDD, and the PDD curve is normalized as the percentage of the maximum dose. Generally, the PDD curve for a high energy photon beam steeply increases to the maximum dose depth in the build-up region due to increasing amounts of scattered photons and secondary electrons. Beyond the maximum dose depth, the PDD curve gently decreases with depth in the build-down region due to attenuation of photons and a decrease in electron fluence. In this test, the maximum PDD values are found to be at a depth of approximatly 15 mm for the 6 MV photon beam in both cases [2,15,18].

In order to maximize therapeutic gain or to minimize radiation hazard, the skin dose is an important factor to consider during radiosurgery treatment. Therefore, the dose distribution near the skin surface should be measured precisely prior to radiosurgery treatments. For measuring skin dose, the dosimeter size is very important and the output signals of dosimeter are normally affected by the thickness of dosimeter [19]. In general, the skin dose measured using the radiochromic film is lower than that measured using a scintillating fiber-based dosimeter or a small ionization chamber due to the relatively small sensing volume of the radiochromic film. It is known that the fiber-optic dosimeter using a scintillating fiber with diameter of 1 mm, skin dose for 6 MV photon beam is higher than 40% [1,2,18].

## 4. Conclusions

In this study, a novel T-shaped fiber-optic phantom-dosimeter system was developed using square scintillating optical fibers, a lens system, and a CMOS image camera. Especially, the square-type optical fibers instead of general round type optical fibers were used to fabricate the T-shaped optical fiber array. The rectangular structure of these square optical fibers enables to minimize the air-gap among the closely packed optical fibers, thereby avoiding the dose measurement errors arising from air-gaps. In order to test the fabricated phantom-dosimeter system, the transverse and longitudinal dose distributions were simultaneously measured with small field sizes. In other words, using a transverse fiber-optic array of the T-shaped fiber-optic phantom, the cross-beam profiles and the beam edge were obtained and PDDs in the build-up region were measured using the longitudinal fiber-optic array. As experimental results, the measured beam profiles were gradually become uneven with increasing depth of the PMMA phantom and the beam edge had a gentle slope with increasing depth. In addition, the maximum dose values for 6 MV photon beam with field sizes of 1 × 1 and 3 × 3 cm^2^ were found to be at a depth of approximately 15 mm and the percentage depth dose of both field sizes were nearly in agreement at the skin dose level.

The proposed integral phantom-dosimeter system offers many advantages over conventional dosimeters in radiosurgery dosimetry. First, the fiber-optic phantom is only composed of the water-equivalent materials, such as scintillating fibers, plastic optical fibers, and PMMA blocks. Therefore, corrections for temperature, pressure, and humidity are not necessary [14,15] and the proposed system makes it possible to measure accurate absorbed dose with minimal perturbation. Second, dosimetry with the scintillating fiber array in the fiber-optic phantom can accurately measure the cross-beam profile and PDD with a high spatial resolution, because each sensitive volume is very small. Finally, the real time and multi-channel measurements capability of the proposed system could reduce the measuring time of high-energy photon beam and provide opportunities for the real-time feedback in quality assurance (QA) of the radiosurgery equipments.

Further studies will be carried out to measure a multi-dimensional dose distribution by using a newly designed phantom-dosimeter system which is only composed of an optical fiber array without PMMA blocks. Based on the results of this study, it is expected that an integral phantom-dosimeter can be developed to accurately measure beam conditions and dose distributions in a small irradiation field prior to conducting stereotactic radiosurgery.
